# A Novel approach of Esthetic Management and preserving Vitality of Dilacerated Permanent Maxillary Lateral Incisor

**DOI:** 10.5005/jp-journals-10005-1354

**Published:** 2016-06-15

**Authors:** Ravindranath C Achary, GR Ravi

**Affiliations:** 1 Reader, Department of Pedodontics, Kids “N” Clips Family Dental Hospital, Kurnool, Andhra Pradesh, India; 2 Reader, Department of Pedodontics, Sudha and Nageswara Rao Siddhartha Institute of Dental Sciences, Chinnoutpalli, Andhra Pradesh, India

**Keywords:** Esthetics, Crown-root dilaceration, Dilaceration, Vitality.

## Abstract

Dilaceration of the permanent tooth usually is a consequence of traumatic injuries to the primary teeth. Although it may appear anywhere in the long axis of the tooth, i.e., crown, cementoenamel junction, or root, most often the root is involved. However, crown dilaceration is a rare condition representing 3% of the total injuries. Maxillary incisors are more susceptible to such injury and affected tooth may either erupt buccally or lingually or remain impacted. Hitherto, the treatment options also differ as per the clinical scenario.

This article proposes a novel technique of restoring esthetic function of the affected permanent maxillary lateral incisor with crown-root dilaceration while preserving the vitality of tooth.

**How to cite this article:** Achary RC, Ravi GR. A Novel approach of Esthetic Management and preserving Vitality of Dilacerated Permanent Maxillary Lateral Incisor. Int J Clin Pediatr Dent 2016;9(2):152-155.

## INTRODUCTION

Traumatic injury to the primary tooth can result in various deformities in the permanent successor. Dilaceration might occur anywhere along the length of the tooth, i.e., the crown, the cementoenamel junction, along the root, or the root apex.^1-3^ Andreasen et al^4-6^ defined dilaceration as the abrupt deviation of the long axis of the crown or root portion of the tooth, which is due to a traumatic nonaxial displacement of already formed hard tissue in relation to the developing soft tissue. Severity of dilaceration usually depends on the relationship of primary root to the developing permanent tooth and the stage of developing permanent tooth gem during the time of injury.^7-9^

Although dilaceration may appear in both permanent and primary teeth, the prevalence is much lower in the later ages. Gender predilection of dilaceration has been reported as 1.6 male to female ratio. Usually, it is known to affect the roots and most commonly of permanent maxillary incisors. Crown dilaceration of a permanent tooth constitutes 3% of all traumatic injuries to developing teeth and is habitually due to intrusion or avulsion of their primary predecessors.^10^ Crown dilacerations with palatal angulation of the crown occur most commonly in maxillary incisors, whereas labial angulation is more common in mandibular incisors.^11,12^

Maxillary incisors when affected may either erupt buccally or lingually or remain impacted. This ectopic eruption of the affected tooth will have a definite effect on the esthetics. Thus, a compromise in the esthetics will be the main reason for an individual to seek treatment. Henceforth, restoration of esthetics will be quite challenging to the clinician and may be at times unique depending on the situation.

## CASE REPORT

A 12-year-old boy reported to the dental clinic with the chief complaint of irregular upper left front teeth. The permanent tooth which erupted was asymptomatic and had altered color and shape. History revealed that the child had an injury at the age of 3 years. As the primary tooth was intruded, it was extracted. Medical history revealed that the child was healthy and had no history of systemic diseases.

**Figs 1A to C:**
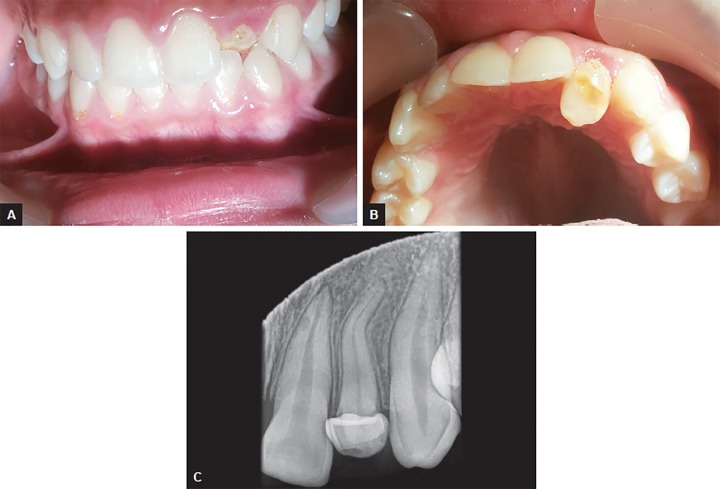
(A) Anterior crossbite in relation to #22, (B) crown dilaceration of #22, (C) periapical radiograph showing crown-root dilaceration of #22

On clinical evaluation, the maxillary left lateral incisor exhibited crown dilacerations affecting incisal and middle third of the crown with an acute bend palatally. The arch form of the maxillary dentition appeared to be in alignment except maxillary left lateral incisor which was in cross-bite (Figs 1A and B). The junction between dilacerated and nondilacerated area on labial and palatal side was yellowish in color and was hypoplastic. The labial surface was pitted and an area of dentin was exposed corresponding to the surface which was in contact with the opposing tooth under occlusion. The palatal surface exhibited white spot lesions which could be attributed to food accumulation.

On radiographic examination, intraoral periapical radiograph (Fig. 1C) showed maxillary left lateral incisor with crown dilaceration affecting incisal and middle third of the crown directed palatally. In addition, even the root was affected with dilaceration at the apical third. The periodontal ligament space was normal along the length of the root with no appreciable periapical changes. Using localization techniques (Same-Lingual Opposite-Buccal technique and occlusal view), it was evident that the coronal portion of the pulp chamber was not extending into the dilacerated portion of the crown. Electric and thermal tests confirmed the vitality of the concerned tooth.

As esthetics was the patient’s prime concern, the proposed treatment had to emphasize on correcting the crossbite, restoring esthetics without compromising the vitality of the tooth. Thus, the treatment planned was in three phases.

- Phase 1―Preserve the vitality of the affected tooth and correct the crossbite.
- Phase 2―Reduction of palatal deflection of the crown and evaluation of any iatrogenic exposure of pulp in due course.
- Phase 3―Restorative treatment with composite resin restoration for esthetics.

After explaining the proposed treatment plan and posttreatment complications to the parents, an informed consent was obtained.

**Fig. 2:**
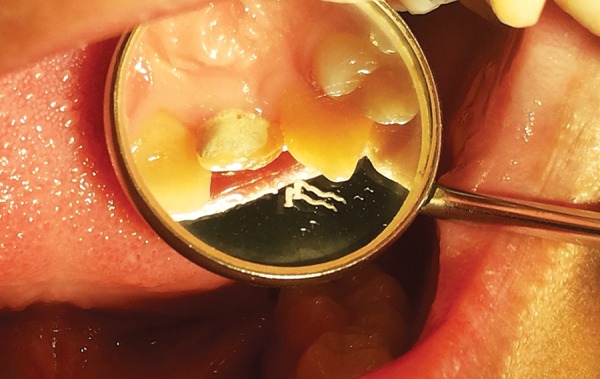
Undercut on the palatal surface being blocked with polycarboxylate restorative material

Phase 1―The main objectives during the first phase were to correct the arch alignment by moving the palatally placed tooth labially and concomitantly prevent pulp exposure during the incremental palatal crown reduction. Labial movement was achieved by modified Hawley’s appliance with double cantilever spring in relation to #22 and posterior bite plane. During this phase of treatment, the path of insertion and removal of Hawley’s appliance and activation of double cantilever spring was found to be difficult due to deflection of crown on the palatal side resulting in an undercut on palatal portion of the crown. To overcome this hindrance, the undercut was blocked with polycarboxylate restorative material which enhanced ease of insertion and removal of appliance (Fig. 2). With regular activation of double cantilever spring, the alignment of affected tooth was achieved within 5 weeks. Once corrected, the 2nd and 3rd phases of treatment were carried out simultaneously.

**Figs 3A and B:**
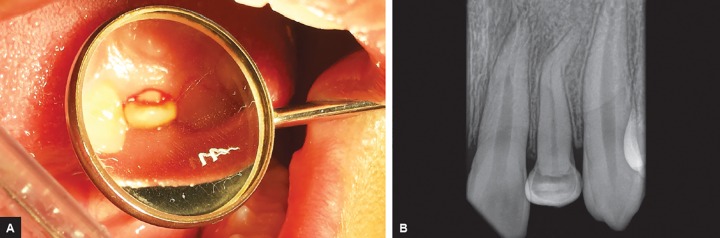
(A) Incremental reduction of palatal surface without any pulpal exposure, (B) periapical radiograph confirming the intact pulp chamber

Phase 2―The incremental reduction of the palatally deflected crown portion was done along the long axis of the tooth so as to maintain enough clearance on palatal and incisal surface for appropriate coronal buildup. There was no iatrogenic pulp exposure and indirect pulp capping done with calcium hydroxide (Dycal) on some amount of leftover dentin (Figs 3A and B).

**Figs 4A and B:**
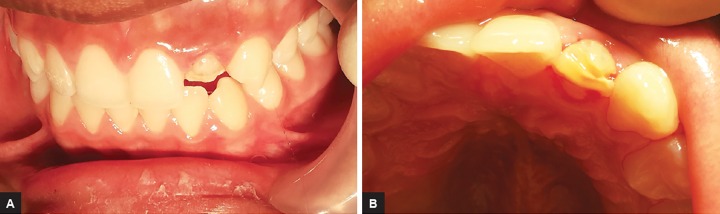
(A) #22 after the correction of anterior cross-bite, (B) placement of retentive grooves on the labial surface of #22

**Fig. 5:**
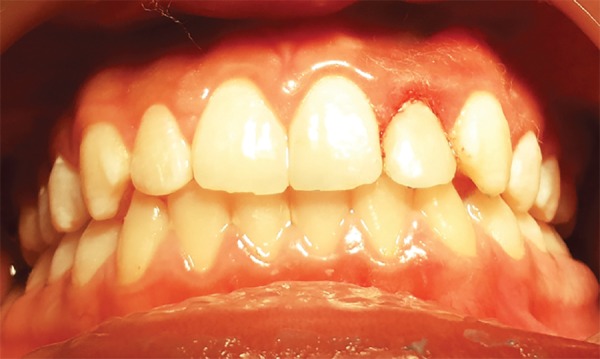
Final restoration of the dilacerated #22

**Fig. 6:**
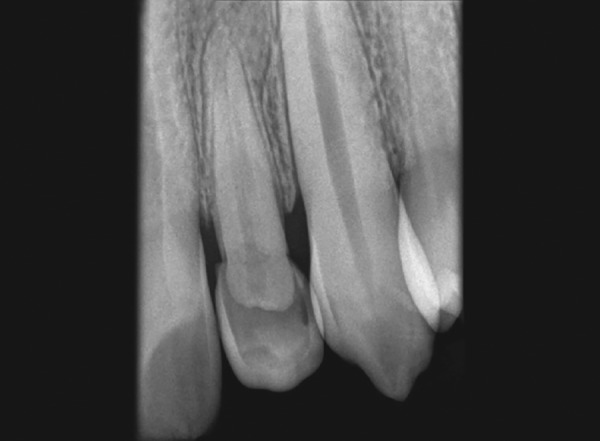
Periapical radiograph of the tooth after 18 months follow-up showing no signs of pathology

Phase 3―Retentive grooves were made on mesiopalatal and distopalatal aspect for additional retention and esthetic composite resin restoration was performed (Figs 4A and B). The final restoration was finished and polished in subsequent session with fine and extrafine disk (Dentsply) and followed up for 18 months (Figs 5 and 6).

## DISCUSSION

Dilaceration is a relatively rare condition wherein only 3% among all kinds of traumatic injuries will result in crown dilacerations. Intrusive or avulsion injuries in the primary dentition can result in dilaceration of permanent successor, at any level of the tooth. The level of dilaceration usually depends on the stage of development of affected tooth at the time of injury. It has been observed that the severity, type, and direction of injury to the primary predecessor will influence the eruption of succedaneous tooth; 50% of them can erupt either normally or labiolingually, while the remaining can remain impacted. In the present case, #22 was erupting palatally and the dilaceration was observed between incisal and middle third of the crown, while root dilaceration was confirmed at apical one-third by periapical radiograph.

Although the initiation of tooth germ of #22 starts *in uetro;* crown completion occurs at the age of 4 to 5 years. In the present case, history revealed that at the age of 3 years, 62 was extracted following intrusive injury. Trauma that occurred during this age might have resulted in crown dilaceration with defective enamel formation leading to enamel hypoplasia at dilacerated area of the tooth.

From the literature, it is clearly known that #22 erupts at the age of 8 to 9 years; root completion is at 11 years and usually during the time of eruption only two-thirds of the root formation is completed. Any insult or trauma to the tooth during this period may result in root dilaceration. In the present case, root dilaceration was observed at the junction of cervical 2/3rd and apical 1/3rd of the root. Although there was no history of injury at the age of 8 to 9 years, clinically #22 was in anterior crossbite. At the junction of dilacerated and nondilacerated area, hypoplastic enamel with dentin exposure was observed. The exposure of dentin could be attributed to the traumatic occlusion with the opposing tooth. This sort of severe occlusal interference during the time of eruption could have resulted in root dilaceration.

Although the priority was to treat the chief complaint by restoring the esthetic and functional element, the prime intension was to preserve the vitality of the pulp during and even after the treatment. So a balanced treatment plan which addressed both the issues was planned and the treatment was carried out in three phases. The electric pulp vitality test was performed to confirm the vitality during each phase of the treatment and even after the treatment at every 6-month intervals for 18 months.

## CONCLUSION

Dilaceration of permanent teeth cannot be ignored for its rarity as it has been one of the main factors responsible for impaction of the involved tooth. Further, it is important to note that dilaceration can result even in the absence of injury to the primary tooth. With variations in clinical and radiographical findings, every case has to be considered unique and treated accordingly. The present case was a humble and novel attempt to balance esthetics, function, and vitality of the crown-root dilacerated tooth.
